# Collection de Memoires sur la Physiologie, la Pathologie, et le Diagnostic

**Published:** 1831

**Authors:** 


### XXII.

**Collection de Memoires sur la Physiologie, la Pathologie, et le Diagnostic. Par P. A. Piorry, M.D., Paris, 1831.**

About three-fourths of this volume are taken up with percussion, by means of the pleximeter, and with physiological experiments on animals, more especially to ascertain the effects of sanguineous depletion. The last fourth of the work is occupied with the diagnostic appearances of the tongue, and the effects of inordinate abstinence on the thoracic and abdominal organs. Of the phenomena presented by the tongue, we gave a concise analysis in our 29th Number (July 1, 1831) and therefore we shall not touch on that subject in the present paper. We shall proceed at once to the memoir "On Abstinence, on Insufficient Alimentation, and their Dangers."

Hypotheses rise and fall; but faithful observations or facts resist the hand of time. No precept has stood this test more fully than that which tells us that 'abstinence is useful in acute diseases.' Men have always a tendency to run into extremes, and carry the most salutary precepts into dangerous excesses. Our author sets out by reminding his readers that the fluids of the body exercise a great influence on the functions of the machine; and that these fluids, more especially the blood, which is the source of all the secretions, are greatly modified, not only by the quality but the quantity of our food. The organs of the body suffer from deficiency as well as from excess of their natural stimuli. In the *former* case, the blood is not carried with sufficient energy towards the brain; and what is curious, there arise, in such circumstances, symptoms very closely resembling cerebral congestion. Pains take place in the muscles not properly supplied with stimulus to activity—the eye gets inflamed in dark places, and often is cured spontaneously by exposure to light—the stomach deprived of sufficient alimentation becomes the seat of severe pains and obstinate vomiting. The author then traces the effects of rigid abstinence (la diète absolue) and insufficient alimentation on the blood and muscles — the heart—the lungs — the digestive organs — and the nervous system.

### I. On the Blood.

There is no doubt, he observes, that the first effects of rigorous and prolonged abstinence is a diminution in the quantity of the circulating fluid. Collard, of Martigny, has made many interesting experiments on this point. The proportion of fibrine diminishes in proportion to the diminution of alimentation; while albumen, on the other hand, augments. The whole volume of blood also diminishes, while the muscles decrease in size and firmness. Fourteen days of "diète absolue" were sufficient to render the muscles of the extremities exceedingly flaccid, wasted, and weak. Their constituent principles are, in fact, absorbed, to repair the loss which the blood necessarily sustains by secretion, &c. The muscles of the trunk experience a similar degree of atrophy, and our author thinks it probable that the muscular pores of the viscera undergo a similar change. The fat, and even the denser tissues of the body must, though more slowly, share the same fate, although there are instances on record where death was produced by starvation, without emaciation. We much doubt these statements. It is, however, to be recollected that increased absorption, in consequence of diminished quality of blood, is not near so great in disease as in health—nor of diseased parts so much as of sound parts. Hence it is that we may starve our patient before we produce the absorption of a morbid growth in any part of the body.

The blood diminishes but little during the first few days of sickness and abstinence, because the various organs furnish materials for the circulation, and so do the drinks taken, and even the air we breathe. But afterwards we see the same phenomena which succeed hæmorrhages. The lips, the tongue, and the conjunctivæ become pale—the veins flatten, and the circulation through them becomes slow—the arteries beat with less force—the heart diminishes in size—the chest returns a clearer sound —and the liver shrinks. The ultimate effects, too, of hæmorrhage and abstinence are the same, debility—disinclination to motion—slowness and torpor of all the functions—tendency to syncope in the erect position—and finally, death, from defect of cerebral excitation. It is thus that the scene terminates, when profound lesions of the alimentary tube obstruct assimilation — when inordinate evacuations carry off the pabulum of life—or where prolonged abstinence cuts off the supply of nutriment from the body. To deprive a patient long of food, is to bleed him largely—and it would often be better to bleed him and give him nourishment at the same time, than to starve him too long. Venesection may be carried to a great extent with safety, when the alimentary tube is in a sound state, and capable of supplying chyle, when nutriment is at length given; but we should be cautious of severe depletion or rigid abstinence, when the primæ viæ are in a different condition; for then we shall find it difficult to re-establish strength when the malady is at an end.

"Would we (says M. Piorry) try to cure phthisis or cancer by starvation? This rigid regimen, by cutting off the supply of nutrition, will only hasten the fatal catastrophe! We dread irritation; but we incur still more dangerous consequences—inanition and its accompaniments. Extreme low diet (la diète absolue) will kill a dog in twenty-five or thirty days; yet we put men on this system for months!"

It is vain, says he, to tell us that the patient takes gum-water, sugared water, emulsions, &c. This is not sufficient alimentation, either for people who are sick, or those who are well. The dogs which Magendie fed on sugar, oil, butter, and gum, died in little more than thirty days of this regimen. If you wish to prolong the days of those who labour under incurable diseases, you must supply the blood with chyle. Do we not see the wound made in lithotomy heal under a nutritive diet, and remain open if we keep the patient too low? How can we expect, then, to heal an internal ulcer by starvation? Let it also be remembered that, in internal ulcerations or malignant diseases of structure, there is an increased absorption, where rigid abstinence is enjoined, and, consequently, a vitiation of the blood. To prevent this, we ought to supply healthy chyle. We see that people who would die in town, get well in the country. No doubt there is a great deal owing to the pure air of the latter situation. But are we to attribute nothing to the increased nourishment which the change of air and scene enables the patient to take? Good wine, says the Doctor, and generous living, are the best tonics. Our estimable Pinel acknowledged that the "VIN D'ARBOIS" saved his life, when medicines failed. It is thus, perhaps, that the Charlatan sometimes succeeds, when the physician, with his rigid diet, sees his patient daily get worse. Our author does not accuse M. Broussais so much as his blind disciples, in starving their patients to death through the chimerical fear of irritation. "I have seen, (says he) patients who had been kept fifteen twenty, thirty days—nay, six or eight weeks, on gum-water; and to whom chicken-broth has been refused, the terror of irritation being so great, that the Doctor trembled at substituting toast-water for the crystal spring." "On other occasions (says he) certainly not numerous, but likely to be so, I have known individuals feeble, pale, and emaciated, condemned to absolute rest, and to live for years in protracted agony (vivre, pendant plusieurs années, dans une longue agonie) on sugar and water, a small quantity of milk, &c." These errors have arisen out of an exaggerated fear of *inflammation*, rather than of irritation—for the *latter* is often increased by too rigid abstinence, debility being the parent of irritability. Sick or well, and especially in chronic maladies, there ought to be allowed a sufficient nourishment for the support of the human machine—and the quantity is to be proportioned to the age and strength of the patient—but more especially according to his former habits and his constitutional idiosyncrasy. The following picture is not imaginary.

"Take a man in perfect health, and submit him to the following regimen. Give him two or three plates of soup maigre, (potages de quelque onces par jour)—eau sucrée for drink—and even add to this an egg. Prohibit bread and wine—and reflect on the consequences of this regimen. The sense of hunger will be only momentarily assuaged during the first few days; but afterwards it will diminish, because this sensation, like all others, is under the dominion of habit. In the course of a week or ten days, the individual will experience debility, will be thinner, and his muscles more flabby. In another week, these phenomena will all be increased—and the stomach will probably become irritable, and even painful. By perseverance in this regimen, the patient (for he is now really ill) will offer all the symptoms of one who has suffered from repeated hæmorrhages."

### II. On Abstinence in Diseases of the Heart.

Under the influence of abstinence, the heart, like all the other muscles, diminishes in volume—and on this fact was founded the treatment of Valsalva. This treatment is indicated in hypertrophy of the ventricles—especially when uncomplicated with contraction of the orifices. Abstinence will have no effect on this last complaint—sometimes a bad effect. Nature strengthens the muscular fibres of the heart, when they have an unnatural resistance to overcome. By diminishing the strength of the parietes we weaken the power of carrying on the circulation, without in any way removing the obstruction. It is not the violent action of the heart which we have so much to dread, as the cause which produces it. The great object, in such cases, is, not so much to lessen the size of the heart, as to proportion the calibre of the arteries, and the orifices of the central organ, to the volume of blood which is in circulation, while, at the same time, we bear in mind that there is a certain force to be sustained in the various other organs of the body. Then it is that repose is necessary, in order that a smaller quantity of blood may suffice for supporting life. To diminish the quantity of the circulating fluids, and impoverish (to use an antiquated expression) the quality of the blood, is the most rational treatment in these diseases. And yet the defect of nutrition, and the general debility, which are the inevitable results, must ultimately terminate fatally. Our author thinks that detractions of blood from the arm, by which the volume is quickly lessened and the embarrassment of the circulation relieved, are better than slow evacuations by leeches, which debilitate, without materially relieving the patient.

It is also to be remembered, that hypertrophy of the heart exists under two very different forms—one, where the muscular structure is firm and resisting—the other where it is soft and flabby. The former state appertains chiefly to youth and robustness of constitution—the *latter* to age and debility. Unfortunately, the diagnosis of these two different conditions is not so firmly established as some pathologists imagine. Auscultation has promised rather more than it has performed. "The *bruits* are very deceptive, percussion is insufficient, and the functional symptoms lead us often astray." Nevertheless, he acknowledges that auscultation, percussion, and a careful observation of symptoms, may enable us to do a great deal in the mitigation of these dreadful afflictions.

In respect to those passive dilatations of the heart, with mollescence of its structure, which we so often meet with, an extreme low diet is not only inefficacious, but absolutely injurious. Even simple dilatation of the heart, whether of the right or left cavities, does not require extreme abstinence — on the contrary, the dilatation is generally increased by such procedure. Where there is reason to believe that the parietes of the organ are soft, we ought to give nourishing diet, and even tonics, especially steel.

"Of the great numbers who have applied to me for palpitation of the heart and difficulty of breathing, and who considered themselves affected with organic diseases of the heart, the greater number of them had no hypertrophy or dilatation — no stethoscopic bruit — no symptom of stricture of the orifices. Many of these, especially medical men, had been put upon vegetable and most debilitating diet; nevertheless they received no benefit from that plan. They went up stairs with great difficulty, and all their symptoms went on increasing in severity. Not perceiving the proofs of organic disease, I changed the plan of treatment, prescribed substantial and plentiful diet, and soon found that the amelioration was progressive and decisive." The author details a remarkable case in illustration.

### III. ON THE DANGERS OF ABSTINENCE IN DISEASES OF THE LUNGS.

In unequivocal inflammation of the lungs or their investing membranes, there can be no question about the necessity of rigid abstinence. But will this hold good, says he "in those congestions of the lungs evidently the result of mechanical causes, of defect of venous action—in those stases of black blood which take place consecutively to diseases of the heart—in old people, and in those who have been long enfeebled by chronic maladies?" I do not, says he, think it will. The principal indications, he observes, which present themselves are, to diminish promptly the quantum of blood circulating through the heart and lungs—and then to stimulate these organs into greater energy. It is not *irritation*, says he, which attracts the blood to the lungs in diseases of the heart. It is the mechanical obstruction to the circulation, and the gravity of the fluid itself, in conjunction with the weakness of the powers which move the blood. Take away, therefore, some blood, to give freedom to the circulation, and then give energy, if possible, to the organs which impel the vital fluid, by generous diet, in small quantities at a time, and watching the effects. This is bold doctrine, even in England, and in France it must be downright heresy. "Yet," says M. Piorry, "it is not without long experience and ample reflection that I have come to these conclusions." This theory rests upon solid and numerous facts. A great number of pulmonic inflammations, coupled with cardiac affections, in old people, were treated at the Salpetriére by blood-letting; but on the succeeding, and sometimes on the very same day, bouillons and light soups were given, occasionally wine. This plan succeeded better than the plan of rigid abstinence.

Regimen, says he, in phthisis pulmonalis, is of the utmost consequence. Broken-down or softened tubercles will not heal on the starving system—nor will crude tubercles be absorbed by that system. Expectoration will not be rendered easier by depriving the patient of alimentary sustenance. On the contrary, the absorption of pus from ulcerations in the lungs will be increased by abstinence, and hectic fever thus kept up—"in short, by starving a phthisical invalid, we add an additional evil to a frightful disease." Pathological anatomy teaches us that ulceration of the intestines is one of the most common causes of debility, and even of death, in phthisis. Vegetable food, which is more difficult of digestion than animal aliment, irritates these ulcerations, and increase the malady. "I am (says M. Piorry) very much deceived, if animal food should not be found necessary and beneficial in a great number of phthisical cases, that are now doomed to asses' milk and farinaceous aliment."

### IV. ON THE EFFECTS OF ABSTINENCE IN DISEASES OF THE DIGESTIVE TUBE.

No practitioner would dream of giving animal, or any kind of full diet, in acute inflammatory affections of the stomach or bowels; but, M. P. observes, there is some difficulty in ascertaining the time when we ought to discontinue the starvation system, when the disease assumes the chronic form. The appetite of the patient is some indication; though it is not always to be depended on. The effects resulting from the process of digestion, and also from the sanguification which ensues, are more safe criteria. Because the tongue is loaded, and the appetite nul, we are not thence to conclude that food is unnecessary. Often, in such a case, the attempt to masticate will recal some goût for victuals, and quickly clean the tongue. Pain in the stomach is not always a counter-indication against food. On the contrary, there are many cases and constitutions, where the epigastric pain is lessened by eating and increased by long fasting. Patients of this kind will throw off slops from their stomachs, and both relish and digest animal food.

The author has pushed his remarks to the diseases of the encephalon, and the effects of rigorous depletion and abstinence in such cases. But our limits are exceeded, and we must close the paper here.

